# Low-dose Spironolactone Combined with ACEIs/ARBs May Reduce Cardiovascular Events in Patients with CKD Stages 3b-5: A Nationwide Population-Based Cohort Study in Taiwan

**DOI:** 10.7150/ijms.103390

**Published:** 2025-02-24

**Authors:** Li-Nien Chien, Po-Jen Hsiao, Chih-Chien Chiu, Wan-Ting Chen, Chih-Jen Cheng, Leon Li-Ming Tsou, Yung-Hsi Kao, Chu-Lin Chou, Te-Chao Fang

**Affiliations:** 1 Institute of Health and Welfare Policy, College of Medicine, National Yang-Ming Chiao Tung University, Taipei, Taiwan.; 2 Graduate Institute of Data Science, College of Management, Taipei Medical University, Taipei, Taiwan.; 3 Division of Nephrology, Department of Internal Medicine, Taoyuan Armed Forces General Hospital, Taoyuan, Taiwan.; 4 Division of Nephrology, Department of Internal Medicine, Tri-Service General Hospital, National Defense Medical Center, Taipei, Taiwan.; 5 Department of Life Sciences, National Central University, Taoyuan, Taiwan.; 6 Division of Infectious Disease, Department of Internal Medicine, Taoyuan Armed Forces General Hospital, Taoyuan, Taiwan.; 7 Division of Infectious Disease, Department of Internal Medicine, Tri-Service General Hospital, National Defense Medical Center, Taipei, Taiwan.; 8 Institute of molecular and cellular biology, National Tsing Hua University, Hsinchu, Taiwan.; 9 Health and Clinical Data Research Center, Office of Data, Taipei Medical University, Taipei, Taiwan; 10 Department of Internal Medicine, The University of Iowa Carver College of Medicine, Iowa City, USA.; 11 Jericho High School, Jericho, Nassau County, New York, USA.; 12 Division of Nephrology, Department of Internal Medicine, School of Medicine, College of Medicine, Taipei Medical University, Taipei, Taiwan.; 13 Taipei Medical University-Research Center of Urology and Kidney, Taipei Medical University, Taipei, Taiwan.; 14 Division of Nephrology, Department of Internal Medicine, Shuang Ho Hospital, Taipei Medical University, New Taipei City, Taiwan.; 15 Division of Nephrology, Department of Internal Medicine, Hsin Kuo Min Hospital, Taipei Medical University, Taoyuan City, Taiwan.; 16 Graduate Institute of Clinical Medicine, College of Medicine, Taipei Medical University, Taipei, Taiwan.; 17 Division of Nephrology, Department of Internal Medicine, Taipei Medical University Hospital, Taipei Medical University, Taipei, Taiwan.

**Keywords:** acute renal failure, acute kidney injury, chronic kidney disease, spironolactone, cardiovascular disease, dialysis, hyperkalaemia

## Abstract

**Background:** ACE inhibitors (ACEIs) and angiotensin receptor blockers (ARBs) are commonly prescribed for hypertension and chronic kidney disease (CKD) management, but they can increase the risk of renal function deterioration and hyperkalaemia. Spironolactone, known for reducing cardiovascular events in CKD patients, faces limited use due to the risk of hyperkalaemia. This study evaluates the potential efficacy and complications of low-dose spironolactone as an adjunct therapy in patients with CKD stages 3b to 5 who are maintained on ACEIs or ARBs.

**Materials and methods:** Hypertensive CKD patients (stages 3b-5) from Taiwan's National Health Insurance Research Database (2012-2016) were selected. Inverse probability treatment weighting (IPTW) was applied to balance baseline characteristics between patients treated with and without spironolactone. In this study, adherence to low-dose spironolactone (25 mg/day) was assessed using the medication possession ratio (MPR) over a continuous 3-month period within the first 12 months after initiation. Multivariate Cox regression models were used to compare clinical outcomes between two groups with MPR ≥80% and MPR <80%. The subgroup including poor adherence (MPR ≥40% and MPR <40%) was also evaluated.

**Results:** Of the 2,623 advanced CKD patients on ACEIs/ARBs and spironolactone, 55.5% (n=1,456) had an MPR ≥80% over a median follow-up of 3.9 years. Post-IPTW, both groups were balanced. Patients with MPR ≥80% showed a lower risk of major adverse cardiovascular events (MACEs; aHR = 0.71, 95% CI = 0.57-0.89), nonfatal myocardial infarction (aHR = 0.54, 95% CI = 0.39-0.75), and heart failure hospitalization (aHR = 0.84, 95% CI = 0.72-0.98). No significant risk was observed for acute renal failure (aHR = 0.87, 95% CI = 0.75-1.02), chronic renal failure (aHR = 0.84, 95% CI = 0.71-1.00), or hyperkalaemia (aHR = 0.86, 95% CI = 0.69-1.07) in the MPR ≥80% group. Patients with MPR ≥40% also showed a lower risk of MACEs (aHR =0.78, 95% CI = 0.62-0.99) and nonfatal MI (aHR = 0.66, CI = 0.47-0.93).

**Conclusion:** In Taiwan, higher adherence to low-dose spironolactone (25 mg/day) in ACEI/ARB-treated patients with CKD stages 3b-5 may reduce cardiovascular disease risk without increasing the risk of renal failure or hyperkalaemia.

## Introduction

In patients with advanced chronic kidney disease (CKD), hypertension is common and usually remains poorly controlled. Spironolactone, a mineralocorticoid receptor antagonist, is recommended as an add-on therapy for hypertension and heart failure (HF) 1-3. Spironolactone reduces cardiovascular events and mortality in HF patients and effectively lowers blood pressure (BP) in hypertensive patients 4-9. Moreover, the addition of spironolactone has a beneficial effect on BP in patients with resistant hypertension 10-13. A double-blind, placebo-controlled, crossover trial in the UK indicated that spironolactone is the most effective add-on medicine for ameliorating resistant hypertension despite the concurrent use of an angiotensin-converting enzyme inhibitor (ACEI), an angiotensin receptor blocker (ARB), a calcium-channel blocker (CCB), or a diuretic 14. Thus, the hypertension guidelines from America, Britain and Europe recommend spironolactone as an add-on therapy when other drugs fail to control BP 15, 16. Spironolactone is effective in patients with HF and CKD, but the risk of adverse events and worsening renal function may be greater in patients with advanced CKD. In addition, spironolactone is associated with an increased risk of adverse effects, including biochemical abnormalities (mainly hyperkalaemia), worsening renal function, and discontinuation of the drug because of anaphylactic reactions or gynaecomastia 17. Therefore, the use of spironolactone in this high-risk population must be closely monitored to achieve clinical benefit.

Patients with hypertension or HF frequently have comorbid advanced CKD, which may alter the efficacy, tolerability, or safety of spironolactone. Spironolactone is a medication that is often used as a diuretic and for treating conditions such as HF, high BP, and certain types of edema. It works by blocking the action of aldosterone, a hormone that causes the kidneys to retain sodium and water. By blocking this hormone, spironolactone helps remove excess fluid from the body and lowers BP. One of the potential side effects of spironolactone is hyperkalaemia, as it reduces the amount of potassium that is excreted by the kidneys. Therefore, CKD patients taking spironolactone are usually advised to monitor their potassium intake to avoid hyperkalaemia 14-16. To date, there is little evidence of the effectiveness and safety of spironolactone in patients with advanced CKD stages (3b-5), especially those with an estimated glomerular filtration rate (eGFR) <30 mL/min/1.73 m^2^. In this study, data from real-world databases in Taiwan were analysed to explore the effectiveness of spironolactone on the risk of major adverse cardiovascular events (MACEs), hospitalization for HF (HHF), acute (ARF) and chronic (CRF) renal failure requiring dialysis, and hyperkalaemia among advanced CKD patients who were treated with ACEIs/ARBs.

## Materials and methods

### Study design and database

This was a retrospective cohort study using data from the National Health Insurance Research Data (NHIRD), a population-based claims database covering almost all healthcare services and drug prescriptions under the regulation of the NHI program in Taiwan. Since 1995, all residents of Taiwan have been required by law to enrol in the NHI, resulting in an over 99% coverage rate 18. We also used the National Death Registry (NDR), a population-based registry for the cause of death, to obtain the death information of all residents. In addition, two datasets can be linked by unique encrypted identifiers under the regulation of the Health and Clinical Science Data Center, Ministry of Health and Welfare in Taiwan. The NHIRD is one of the highest-quality databases of its type in the world and has been widely used for longitudinal cohort studies, including several of our previous investigations 19-30.

### Study cohort

First, patients were included if they were enrolled in the pre-end-stage renal disease (pre-ESRD) pay-for-performance (P4P) program between 2012 and 2016, a patient care and education program involving health management of high-risk groups to improve healthcare and delay the onset of ESRD and dialysis initiated in November 2006 31, 32. In this study, all CKD patients received their regular medications such as cardiovascular drugs and antidiabetic drugs. The eGFR was more commonly calculated using the abbreviated Modification of Diet in Renal Disease (MDRD) Study equation in Taiwan. Patients were eligible for the program if they met the following criteria: CKD stage 3b, 4, or 5 with eGFRs of 30-44.9, 15-29.9, or <15 mL/min/1.73 m^2^, respectively; and proteinuria, defined as daily urinary protein levels of >1000 mg or a urine protein/creatinine ratio of >1000 mg/g. In addition, multidisciplinary medical teams, including nephrologists, health education nurses, and nutritionists, provided comprehensive medical assessments, laboratory examinations, and patient education every three months. Those enrolled in the program were also cared for according to applicable clinical guidelines at different stages of CKD 33. The date of initial enrolment in the program was treated as the index date.

We selected patients who were diagnosed with hypertension and excluded those who 1) were aged less than 20 years or had missing sex information, 2) had a catastrophic illness (including cancer) other than dialysis during the overall study period, or 3) had a history of dialysis or liver cirrhosis before the index date. Finally, we limited inclusion to patients treated with ACEIs/ARBs within three months after the index date because we aimed to examine the effectiveness of spironolactone as an add-on therapy to ACEIs/ARBs, which are the preferred agents for treating advanced CKD patients with hypertension. We also used a three-month window to calculate medication compliance. Thus, patients who experienced outcomes within that window were also excluded.

### Study variables

The identification of low-dose (25 mg per day) spironolactone use was based on prescription claims from the NHIRD. Patients were defined as spironolactone users if they received a pharmacy claim for spironolactone in combination with ACEIs/ARBs within three months of the start of the study. We used the medication possession ratio (MPR) to measure compliance with spironolactone treatment. In this study, the MPR represents the ratio of days covered by medication supply across all prescription fills within a specified time interval (continuous 3-month use during the 12 months following the index date). In this study, if a participant does not continue their prescription for more than 90 days during the follow-up period, they will be considered "censored" and will exit the study group, and we will no longer continue observing them. The MPR was further classified into the categories, with <40% as the lower bound and ≥80% as the upper bound. An MPR of 80% is a reasonable threshold for compliance, as it suggests very few days without the drug on hand and, consequently, fairly continuous medication use 34.

### Outcome definition

The outcomes of interest were MACEs, HHF, requiring dialysis, and hyperkalaemia during the follow-up periods. The definition of MACEs was a composite of nonfatal ischaemic stroke (IS), nonfatal myocardial infarction (MI), and cardiovascular death. The occurrence of stroke, myocardial infarction, or HHF was indicated by a discharge record from the NHIRD. ARF requiring dialysis was defined as a payment code for dialysis. If a patient continuously had payment codes for at least three months, the patient was regarded as having CRF. Moreover, the occurrence of hyperkalaemia was defined as a diagnosis of hyperkalaemia after the index date. Finally, all the patients were followed up from the index date to the date of outcome, death or the study end date of December 31^st^, 2018.

### Covariates

The covariates included sex, age, CKD stage, ACEI/ARB MPR, history of hospitalization, comorbidities, medication use, Charlson comorbidity index (CCI), and CHA_2_DS_2_-VASc score (congestive HF, hypertension, age ≥75 years, diabetes mellitus (DM), stroke/transient ischaemic attack, vascular disease, age 65 to 74 years, sex category [female]). Comorbidities associated with the risk of adverse cardiovascular outcomes were defined only if patients had at least two diagnostic claims within one year before the index date. For medication use, patients who had received the medication continuously for at least three months within a year were considered to have a specific prescription. We also used the CCI and CHA_2_DS_2_-VASc score to adjust for the severity of comorbidities.

### Statistical analysis

Several factors might be associated with the effectiveness of spironolactone, resulting in a significant difference in baseline characteristics between the two groups. Thus, we used inverse probability treatment weighting (IPTW) to adjust the imbalance. IPTW, a method based on propensity scoring, was used to balance baseline variables without loss of samples. IPTW is also an appropriate method for estimating treatment effects on time-to-event outcomes 35. The stabilized IPTW was weighted for each patient after the propensity score was generated. This method has been widely adopted in many observational studies. The standardized mean difference (SMD) is presented, and SMD >0.1 indicates nonnegligible differences between groups.

The incidence per 100 person-years of outcomes was calculated via Kaplan‒Meier estimation. A Cox proportional hazards regression model was used to evaluate the hazard ratio (HR) for risk outcomes associated with spironolactone (25 mg/day), after controlling for demographic and clinical factors 36. Patients who died, were lost to follow-up, or were discharged before the event of interest occurred during the follow-up period were censored. Subgroup analysis was also performed by age group, DM status, and CHA_2_DS_2_-VASc score. None of the models violated the assumption of proportional hazards. All analyses were performed via SAS/STAT 9.2 (SAS Institute Inc., Cary, NC, USA). P<0.05 was considered to indicate statistical significance.

## Results

**Figure 1:**
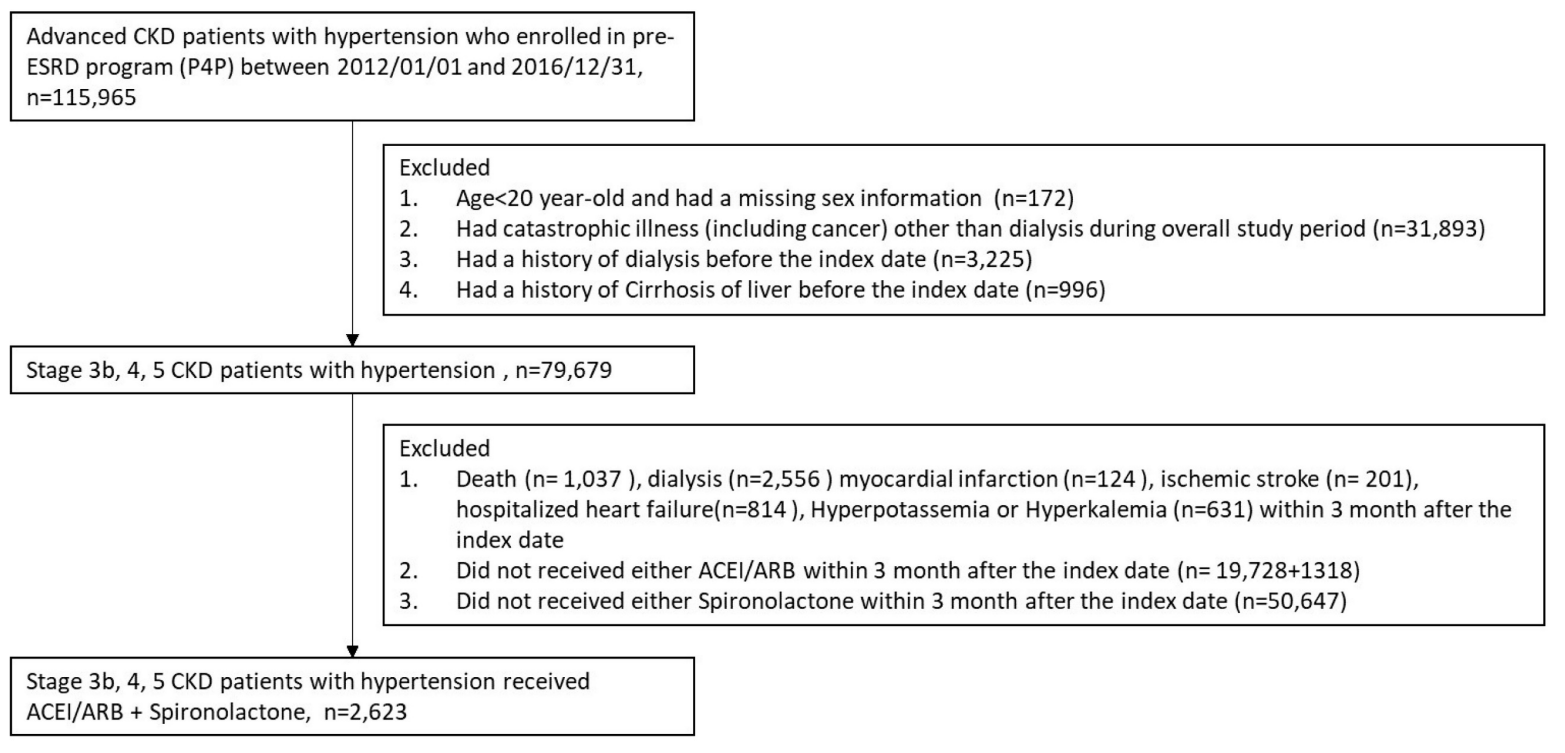
Patient selection process.

The patient selection process is shown in detail in Figure 1. Close to half of the 115,965 advanced CKD patients with hypertension who met the inclusion criteria were eligible for the analyses in this study, as shown in Figure 1. Ultimately, a total of 2,623 participants with CKD stages 3b, 4, and 5 and hypertension treated with ACEIs/ARBs plus spironolactone were enrolled and divided into 2 groups: patients with a spironolactone MPR <80% (n = 1,167) and those with a spironolactone MPR ≥80% (n = 1,456) (see Table 1 for the baseline characteristics of the study cohort). After IPTW, there were no differences in age, sex, CKD stage, MPR, CCI, CHA_2_DS_2_-VASc score, comorbidities, or medication use between the two groups, except during the follow-up period.

Table 2 presents the incidence (per 100 person-years) and adjusted HR (aHR) of cardiovascular and dialysis events for the MPR <80% and ≥80% groups. The incidences of MACEs were 4.84 and 3.43 per 100 person-years for patients with an MPR of <80% and ≥80%, respectively, with an aHR of 0.71 (95% confidence interval [CI] of 0.57-0.89). Differences in nonfatal MI (aHR = 0.54, CI = 0.39-0.75) and HHF (aHR = 0.91, CI = 0.72-0.98) were also detected between the two groups. The result of nonfatal IS showed no significance between the two groups (aHR = 0.83, CI = 0.59-1.16). Moreover, patients with an MPR ≥80% did not experience an increased risk of ARF requiring dialysis, CRF requiring dialysis, or hyperkalaemia.

**Figure 2:**
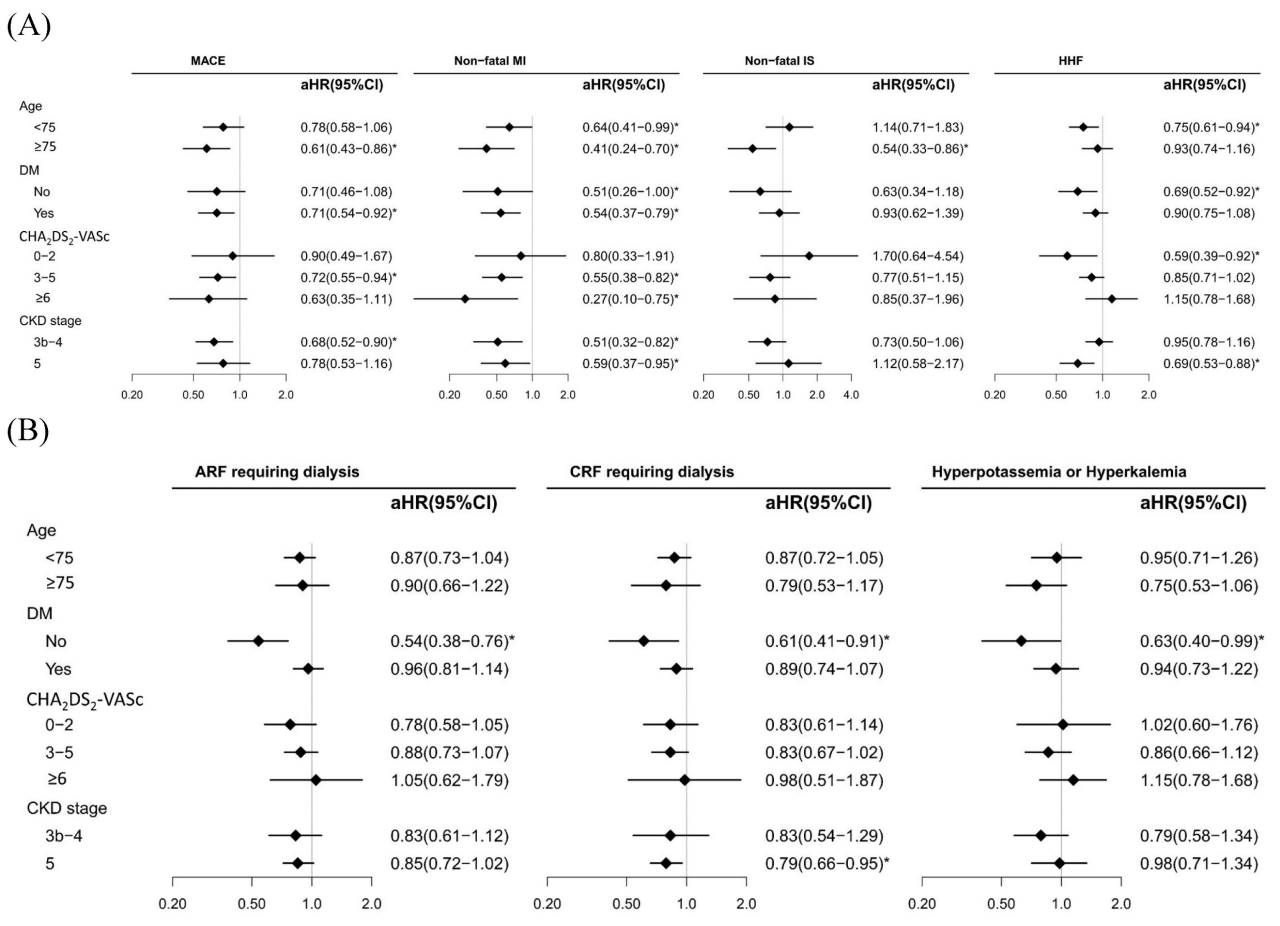
Subgroup analysis for adjusted hazard ratios (aHRs) of cardiovascular (A) and dialysis events (B) between spironolactone MPR groups (<80% (ref.) and ≥80%) in patients with CKD stages 3b-5 treated with ACEIs/ARBs.

Figure 2A shows the subgroup analyses (age, DM, CHA_2_DS_2_-VASc score, and CKD stages 3b-4 and 5) for the benefits and risks associated with spironolactone use, with MPRs <80% (reference) and ≥80% for cardiovascular and dialysis events. For elderly individuals (≥75 years old), our data revealed a lower risk of MACEs, nonfatal MI, and nonfatal IS; however, a lower risk of HHF was observed in patients aged <75 years. DM status was associated with a decreased risk of MACEs and nonfatal MI, whereas non DM status was associated with a reduced risk of HHF. With respect to HHF severity, we utilized the CHA_2_DS_2_-VASc; a mild score (0-2) was associated with a decreased risk of HHF, and an advanced score (3-5 and ≥6) was associated with an increased risk of nonfatal MI. Finally, compared with CKD stage 5, CKD stages 3b-4 were associated with a lower risk of MACEs and nonfatal MI. The study results also demonstrated that there was no significant risk of ARF, CRF, and hyperkalaemia in patients who maintained a MPRs of 80% or higher (Figure 2B). Specifically, the adjusted hazard ratio (aHR) for ARF requiring dialysis was 0.87 with a 95% confidence interval (CI) ranging from 0.75 to 1.02. Similarly, the risk of CRF requiring dialysis was also not significantly elevated, with an aHR of 0.84 and a 95% CI of 0.71 to 1.00. Furthermore, the incidence of hyperkalaemia was not significantly higher in the high adherence group, with an aHR of 0.86 and a 95% CI of 0.69 to 1.07.

Table 3 presents the incidence (per 100 person-years) and adjusted HR (aHR) of cardiovascular and dialysis events for the MPR <40% and ≥40% groups. The incidences of MACEs were 4.77 and 3.72 per 100 person-years for patients with an MPR of <40% and ≥40%, respectively, with an aHR of 0.78 (95% confidence interval [CI] of 0.62-0.99). Differences in nonfatal MI (aHR = 0.66, CI = 0.47-0.93) and HHF (aHR = 0.91, CI = 0.77-1.07) were also detected between the two groups.

## Discussion

In this study, we are the first to explore the associations between low-dose spironolactone use and the risk of cardiovascular events and dialysis in advanced CKD patients treated with ACEIs/ARBs on the basis of real-world data in Taiwan. After adopting IPTW, controlling for covariates, and performing subgroup and sensitivity analyses, our main findings were as follows. (1) Low-dose spironolactone with an MPR ≥80% was associated with a decreased risk of cardiovascular events (MACEs, nonfatal MI, and HHF) and no additional risk of dialysis events (ARF requiring dialysis, CRF requiring dialysis, and hyperkalaemia). (2) Subgroup analyses revealed a decreased risk of MACEs and nonfatal MI in elderly individuals (≥ 75 years old) and DM patients and a decreased risk of HHF in younger individuals (< 75 years old), non-DM patients, and patients with a mild CHA_2_DS_2_-VASc score.

(3) Even when spironolactone with an MPR ≥40% was used, there was still a benefit in terms of MACEs and nonfatal MI. Low-dose spironolactone with an MPR ≥40% also had a protective effect in reducing the risk of MACEs and nonfatal MI. Similar to the MPR ≥80% group, patients with an MPR ≥40% did not experience an increased risk of ARF requiring dialysis, CRF requiring dialysis, or hyperkalaemia.

The use of spironolactone for heart disease treatment in patients with advanced CKD continues to be a challenge in clinical practice. According to a meta-analysis of randomized controlled trials (RCTs; N = 829), compared with the control group, ESRD patients with or without HF who were treated with spironolactone or eplerenone had reduced risks of cardiovascular (RR: 0.34; 95% CI: 0.15 to 0.75) and all-cause (RR: 0.40; 95% CI: 0.23 to 0.69) death 37. Furthermore, a subgroup analysis from the TOPCAT trial, an international, double-blind RCT of spironolactone use in patients older than 50 years of age with symptomatic HFpEF (left ventricular ejection fraction ≥45%),^17^ revealed that patients (N=1767) on spironolactone had a decreased risk (HR 0.82, 95% CI: 0.69-0.98) of cardiovascular events (cardiovascular death, HHF, and aborted cardiac arrest); however, the TOPCAT trial did not include patients with more severe kidney disease (eGFR <30 mL/min/1.73 m^2^ or serum creatinine ≥2.5 mg/dL).

Our results regarding the effects of low-dose spironolactone use with an MPR ≥80% on lowering the risk of cardiovascular events (MACEs, nonfatal MI, and HHF) were similar to some other clinical studies in the Asian and Europe populations 38-40. For example, in a single-centre, observational, retrospective, registry-based clinical study of 200 patients with acute MI and CKD (eGFR <60 mL/min/1.73 m^2^) who were treated with spironolactone, Qu *et al.* reported a reduced risk of both all-cause mortality (HR: 0.389; 95% CI: 0.276-0.548; p < 0.001) and readmission (HR: 0.664; 95% CI: 0.522-0.846; p = 0.004) after 30 months of follow-up. Additionally, compared with high-dose spironolactone use (more than 40 mg; HR: 0.429; 95% CI: 0.199-0.925; p = 0.007), low-dose spironolactone use (no more than 40 mg) was associated with a lower risk of all-cause mortality (HR: 0.309; 95% CI: 0.228-0.418; p < 0.001) 38. In addition, the results from the MiREnDa trial (an RCT) and experiments on mice subjected to subtotal nephrectomy and cholecalciferol treatment supported the benefit of spironolactone treatment in ameliorating CKD-associated vascular calcification 39. Moreover, in an RCT that included haemodialysis patients, spironolactone treatment at a dose of 12.5 to 25 mg for 6 months was associated with the regression of left ventricular hypertrophy 40. Recent evidence has demonstrated that potassium binders can reduce the incidence of hyperkalaemia. The related guidelines address key areas, including monitoring, dietary restrictions, the use of potassium binders, and the concurrent prescription of renin‒angiotensin‒aldosterone system (RAAS) inhibitors. However, further research is needed to determine whether reduced-potassium diets or potassium binder therapy may improve patient-centred outcomes, especially in patients with advanced stages of CKD 41. Although our data revealed the effectiveness and safety of spironolactone treatment in patients with advanced CKD, further prospective RCTs are needed to clarify the effect of spironolactone on cardiovascular events in this population.

The effects of spironolactone treatment on the risk of hyperkalaemia, ARF or acute kidney injury (AKI, acute deterioration of renal function) and the need for emergency dialysis in patients with advanced CKD remain unclear. In the TOPCAT Americas study (n = 1,767) on safety outcomes, including hyperkalaemia, worsening kidney function, and permanent drug discontinuation, the risk of safety outcomes was greater for patients with severe kidney dysfunction (eGFR <45 ml/min/1.73 m^2^: HR, 1.99; 95% CI, 1.62-2.45; p < 0.001) than for patients with relatively preserved eGFRs (≥60 ml/min/1.73 m^2^) during the 4-year follow-up 17. This finding suggested an increased risk for intolerance to spironolactone with decreasing renal function. These data focus on the importance of close laboratory monitoring in patients with HFpEF and an eGFR less than 45 mL/min/1.73 m^2^ who are treated with spironolactone. Furthermore, meta-analyses revealed that the addition of spironolactone to an ACEI or an ARB was associated with an increased risk of side effects, mainly a two- to threefold greater risk of hyperkalaemia 42-44.

Feniman-De-Stefano *et al.* reported that spironolactone treatment at a dose of 12.5 to 25 mg for 6 months was not associated with the risk of hyperkalaemia in haemodialysis patients 40. The safety of spironolactone in a randomized, placebo-controlled study of dialysis-dependent ESRD patients showed a dose-dependent increase related to the risk of hyperkalaemia, particularly when spironolactone was prescribed at a dosage of 50 mg/d. In this small study, spironolactone was found to be safe for well-monitored maintenance haemodialysis patients, though it did not significantly impact cardiovascular outcomes 45. Rajagopalan *et al.* recently reported that spironolactone could reduce the progression of atherosclerosis in diabetic patients with advanced stages of CKD. These RCT results from the United States are also compatible with our study findings 46. Our study results revealed low-dose spironolactone treatment had no additional risk of adverse events regarding dialysis or hyperkalaemia. We propose that these results may be influenced by potassium-restricted diets and drug dosages. In Taiwan, the pre-ESRD program has been effectively implemented due to the high prevalence of ESRD. A low-potassium diet is often recommended for patients in the pre-ESRD program to help manage potassium levels and reduce the risk of complications. Various clinical factors, such as sample size, follow-up duration, racial differences, low-potassium diets, drug dosages, and patient compliance, may impact the clinical outcomes. Long-term benefit-risk profile of low-dose spironolactone treatment in the patients with advanced CKD and ESRD needs further investigation. Finerenone is a new non-steroidal mineralocorticoid receptor antagonist used primarily in the treatment of CKD associated with type 2 DM. It works by blocking the effects of aldosterone and can further decrease kidney damage, fibrosis, and cardiovascular complications. From FIDELIO-DKD and FIGARO-DKD Trial, finerenone has been shown to reduce albuminuria, slow the progression of kidney disease, lower the risk of hyperkalemia and cardiovascular events such as heart failure and stroke in patients with diabetic kidney disease 47-50.

Thiazide and thiazide-like diuretics are widely prescribed in clinical practice. It was believed that these agents would lose effectiveness in patients with the GFR below 30 mL/min/1.73 m² previously 51. Over time, this notion became widely accepted, and this threshold has long been cited in medical textbooks and guidelines to contraindicate thiazides in patients with advanced stages of CKD. The 2017 American College of Cardiology/American Heart Association (ACC/AHA) guidelines for hypertension management advise against using thiazides in patients with GFR <30 mL/min/1.73 m² 52. Similarly, the 2018 ESC/ESH Hypertension Guidelines state that thiazides and thiazide-like agents are less effective in patients with an eGFR <45 mL/min/1.73 m² and are ineffective at eGFR <30 mL/min/1.73 m² 53. Therefore, thiazides are used less frequently among these patients. To avoid the selection bias, baseline medications did not include this drug in our study. However, recent data supporting cardiovascular risk reduction have renewed interest in the use of thiazides in the patients with advanced stages of CKD. These findings should spur new prospective randomized trials and spark discussions, particularly about upcoming hypertension guidelines in CKD patients 54, 55.

### Strengths and limitations

This study is the first to use the MPR to assess the effects of low-dose spironolactone on cardiovascular events, emergency dialysis, and hyperkalaemia in Taiwanese patients with advanced stages of CKD. In our nationwide population-based cohort study, we observed that a low dose of 25 mg spironolactone per day combined ACEIs/ARBs use was safe in patients with CKD stages 3b-5. Our study results revealed that low-dose spironolactone was not associated with ARF or CKD requiring dialysis in these patients. However, there are several limitations in this investigation. First, in observational studies, confounding by indication has often been an intractable threat to validity because patients with poor prognoses are more likely to be treated aggressively. Second, we observed that the patients who were treated with spironolactone had more comorbidities and greater medication use (shown in Table 1); thus, confounding by indication was found. We then adopted IPTW, multivariable regression models, and patient subgroups to improve the validity of the findings in this study; however, no adjustment methods could fully resolve the bias. Therefore, the results of the present study should be interpreted with caution. Moreover, we used data from reimbursement claims; consequently, we could not obtain data on BP, body mass index, or lifestyle. Magnesium levels rise as kidney function declines. Hypermagnesemia is a predictor of cardiovascular events and all-cause mortality in individuals with reduced renal function 56. Associated laboratory data could be investigated in the future study. Finally, the information was derived from individuals of Asian descent, and therefore, generalizability of the findings may be limited to other different races or ethnicities.

## Conclusion

This cohort study revealed that low-dose spironolactone treatment is associated with a reduced risk of cardiovascular events (MACEs, nonfatal MI, and HHF) and no change in the risk of adverse events (emergent dialysis and hyperkalaemia) in patients with stages 3b-5 CKD treated with ACEIs/ARBs. Moreover, we believe that prospective randomized trials further investigating these effects are warranted.

## Figures and Tables

**Table 1:** Baseline characteristics of late-CKD patients treated with AECIs/ARBs + spironolactone before and after IPTW.

Spironolactone, MPR	Before IPTW			After IPTW		
	MPR < 80%n = 1,167, %	MPR ≥ 80%n = 1,456, %	SMD	MPR < 80%n = 1,167, %	MPR ≥ 80%n = 1,456, %	SMD
Male	54.4	56.2	0.036	54.3	54.2	< 0.001
Age (years), [mean, SD]	[69.0, 14.1]	[68.1, 14.1]	0.068	[68.6, 14.1]	[68.8, 13.9]	0.015
20-44	5.2	6.6	0.058	5.8	5.8	< 0.001
45-64	30.6	33	0.053	31.6	31.2	0.008
65-74	21.6	21.8	0.004	21.9	21.7	0.005
≥ 75	42.6	38.6	0.081	40.7	41.3	0.012
Stage						
3b, 4	65.9	78.8	0.293	72.6	73.3	0.017
5	34.1	21.2	0.293	27.4	26.7	0.017
AECIs/ARBs, MPR [mean, SD]	[0.8, 0.3]	[0.9, 0.2]	0.517	[0.9, 0.2]	[0.9, 0.2]	0.012
MPR ≥ 80%	65.3	89.3	0.598	78.3	78.2	0.001
MPR 40-80%	21.3	6.4	0.441	13.3	13.3	0.002
MPR < 40%	13.5	4.3	0.325	8.4	8.4	0.001
CCI [mean, SD]	[4.1, 1.9]	[4.0, 1.8]	0.071	[4.0, 1.9]	[4.0, 1.8]	0.004
CHA_2_DS_2_-VASc [mean, SD]	[3.7, 1.4]	[3.7, 1.5]	0.054	[3.7, 1.4]	[3.7, 1.5]	0.005
0-2	20.3	26.2	0.139	20.8	24	0.078
3-5	68.6	62.5	0.129	67.5	63.8	0.078
≥ 6	11.1	11.3	0.009	11.8	12.2	0.014
History of hospitalization						
AMI	1.1	1.6	0.040	1.3	1.5	0.017
Heart failure	9.9	11.5	0.049	10.8	10.6	0.005
Stroke	6.4	4.7	0.077	5.5	5.8	0.011
Comorbidities						
Diabetes mellitus	64.3	64.1	0.004	65.2	65.1	0.003
IHD	28.5	33.4	0.106	31.4	31.5	< 0.001
Atrial fibrillation	5.6	7.1	0.062	6.4	6.5	0.001
Hyperlipidaemia	48.1	47.9	0.004	47.8	47.9	< 0.001
PVD	3.3	3	0.022	3.2	3	0.009
COPD	17.5	16	0.040	17.2	17.1	0.003
CLD	5.3	3.8	0.070	4.7	4.6	0.004
Dementia	4.3	4.3	0.001	4	4.3	0.015
Medication use						
Clopidogrel	3.5	4.2	0.035	3.8	3.9	0.007
Dipyridamole	6	4	0.093	5	4.9	0.008
Warfarin	1.5	2.7	0.090	2.3	2.2	0.006
ACEIs/ARBs	42.2	40.6	0.032	41.3	41.2	0.003
Loop diuretics	29.2	28	0.028	29.3	29.2	0.003
Beta-2 blockers	23	20.9	0.049	21.8	21.9	0.003
CCBs	28.2	22.8	0.124	25.5	25.5	< 0.001
Antiplatelet drugs	24.7	23.3	0.033	24.8	24.7	0.002
Statins	16.9	14.8	0.056	16.2	15.9	0.009
NSAIDs	26.5	21.2	0.124	24.1	24.2	0.002
Metformin	8.3	6.5	0.068	7.6	7.4	0.008
Thiazolidinedione	3.1	1.9	0.079	2.3	2.1	0.015
Sulfonylureas	12.8	11.9	0.027	12.6	12.5	0.003
AGIs	5.1	4.1	0.045	4.7	4.7	0.002
DPP4is	12.4	10.9	0.049	11.6	11.3	0.008
Insulin	11.2	10.3	0.030	11	10.9	0.002
Follow-up period (years) [mean, SD]	[3.5, 1.9]	[3.9, 1.9]	0.216	[3.5, 1.9]	[3.9, 1.9]	0.174

Abbreviations: ACEIs/ARBs: angiotensin-converting enzyme inhibitors/angiotensin receptor blockers; AGIs: alpha-glucosidase inhibitors; AMI: acute myocardial infarction; CCBs: calcium channel blockers; CCI: Charlson comorbidity index; CKD: chronic kidney disease; CLD: chronic liver disease; COPD: chronic obstructive pulmonary disease; DPP4is: dipeptidyl peptidase 4 inhibitors; IHD: ischaemic heart disease; IPTW: inverse probability of treatment weighting; NSAIDs: nonsteroidal anti-inflammatory drugs; PVD: peripheral vascular disease; SD: standard deviation; SMD: standardized mean difference.

**Table 2:** The incidence (per 100 PY) and adjusted HR of cardiovascular and dialysis events between spironolactone MPR groups (< 80% and ≥ 80%) in patients with CKD stages 3b-5 treated with ACEIs/ARBs.

Outcomes	No. of events	PY	Incidence (95% CI)	Adjusted^*^ HR (95% CI)	P value
MACEs					0.003
MPR < 80%	184	3,806	4.84 (4.19-5.59)	1.00 (Ref.)	
MPR ≥ 80%	183	5,329	3.43 (2.95-3.95)	0.71 (0.57-0.89)	
Nonfatal MI					< 0.001
MPR < 80%	94	3,942	2.39 (1.95-2.92)	1.00 (Ref.)	
MPR ≥ 80%	71	5,489	1.29 (1.03-1.63)	0.54 (0.39-0.75)	
Nonfatal IS					0.264
MPR < 80%	74	3,937	1.88 (1.50-2.36)	1.00 (Ref.)	
MPR ≥ 80%	84	5,456	1.54 (1.23-1.89)	0.83 (0.59-1.16)	
HHF					0.025
MPR < 80%	353	3,369	10.47 (9.41-11.60)	1.00 (Ref.)	
MPR ≥ 80%	412	4,809	8.57 (7.78-9.44)	0.84 (0.72-0.98)	
ARF requiring dialysis					0.077
MPR < 80%	369	3,279	11.25 (10.14-12.43)	1.00 (Ref.)	
MPR ≥ 80%	452	4,690	9.64 (8.79-10.57)	0.87 (0.75-1.02)	
CRF requiring dialysis					0.051
MPR < 80%	295	3,334	8.86 (7.90-9.92)	1.00 (Ref.)	
MPR ≥ 80%	354	4,793	7.38 (6.64-8.18)	0.84 (0.71-1.00)	
Hyperkalaemia					0.181
MPR < 80%	178	3,735	4.76 (4.09-5.49)	1.00 (Ref.)	
MPR ≥ 80%	207	5,148	4.02 (3.49-4.59)	0.86 (0.69-1.07)	

Abbreviations: ARF: acute renal failure; CRF: chronic renal failure; CI: confidence interval; HHF: hospitalized heart failure; HR: hazard ratio; IS: ischaemic stroke; MACEs: major adverse cardiac events; MI: myocardial infarction; PY: person-year; Ref.: reference group.

**Table 3:** The incidence (per 100 PY) and adjusted HR of cardiovascular and dialysis events between spironolactone MPR groups (< 40% and ≥ 40%) in patients with CKD stages 3b-5 treated with ACEIs/ARBs.

Outcomes	No. of events	PY	Incidence (95% CI)	Adjusted^*^ HR (95% CI)	P value
MACEs					0.040
MPR < 40%	122	2,564	4.77 (3.99-5.68)	1.00 (Ref.)	
MPR ≥ 40%	245	6,570	3.72 (3.28-4.21)	0.78 (0.62-0.99)	
Nonfatal MI					0.017
MPR < 40%	61	2,658	2.31 (1.79-2.95)	1.00 (Ref.)	
MPR ≥ 40%	104	6,773	1.53 (1.25-1.84)	0.66 (0.47-0.93)	
Nonfatal IS					0.114
MPR < 40%	54	2,636	2.05 (1.57-2.67)	1.00 (Ref.)	
MPR ≥ 40%	104	6,757	1.54 (1.26-1.85)	0.76 (0.53-1.07)	
HHF					0.243
MPR < 40%	231	2,272	10.18 (8.94-11.57)	1.00 (Ref.)	
MPR ≥ 40%	533	5,905	9.03 (8.29-9.83)	0.91 (0.77-1.07)	
ARF requiring dialysis					0.023
MPR < 40%	261	2,196	11.88 (10.49-13.37)	1.00 (Ref.)	
MPR ≥ 40%	560	5,773	9.70 (8.93-10.54)	0.83 (0.71-0.97)	
CRF requiring dialysis					0.013
MPR < 40%	210	2,223	9.44 (8.21-10.77)	1.00 (Ref.)	
MPR ≥ 40%	439	5,904	7.44 (6.77-8.17)	0.80 (0.67-0.95)	
Hyperkalaemia					0.658
MPR < 40%	107	2,526	4.22 (3.47-5.08)	1.00 (Ref.)	
MPR ≥ 40%	278	6,357	4.38 (3.89-4.92)	1.06 (0.83-1.34)	

Abbreviations: ARF: acute renal failure; CRF: chronic renal failure; CI: confidence interval; HHF: hospitalized heart failure; HR: hazard ratio; IS: ischaemic stroke; MACEs: major adverse cardiac events; MI: myocardial infarction; PY: person-year; Ref.: reference group.
